# Causal Graphical Models and Their Applications

**DOI:** 10.3390/e28040405

**Published:** 2026-04-03

**Authors:** Luis Enrique Sucar, David Danks

**Affiliations:** 1 Instituto Nacional de Astrofísica, Óptica y Electrónica (INAOE), Santa María Tonantzintla, Puebla 72840, Mexico; 2 Department of Philosophy, School of Data Science, University of Virginia, Charlottesville, VA 22904, USA; danks@virginia.edu

## 1. Introduction

The study of causality [1] examines the relations and regularities found in a given environment that are stronger than merely probabilistic (or associative) relations [2], in the sense that a causal relation allows for evaluating a change in the consequence given a change in the cause. Causation is crucial not only for predictive accuracy, but also for control, explanation, and intervention [3]. Causality is central to much of the scientific enterprise and even everyday life, and there has been increasing interest in recent decades in causal modeling, inference, and reasoning. This research has historically fallen into two different traditions, causal graphical models [4,5] and potential outcomes [6,7]. The former provides excellent interpretability and characterization of structural properties, while the latter focuses more on parameter estimation and quantitative causal modeling. These two approaches are equally powerful in terms of causal representation and inference [8] and subsume other approaches [9], but highlight different information and methods [10]. This Special Issue focuses on cutting-edge research with causal graphical models.

Causal models provide a deeper understanding of a domain by identifying the direct and indirect causes of certain events, in contrast with associative models, including most neural/deep network approaches. These representations enable us to perform types of reasoning that are not possible, at least in a direct way, with associative models [11]: (i) *interventions*, where we want to find the effects of an external agent or event forcing a variable to a specific value (in contrast with merely observing that variable’s value), and (ii) *counterfactuals*, where we want to reason about what would have happened if certain information had been different from what actually happened. Both types of causal reasoning are closely connected with explanations, since an effect can be explained by its causes, regardless of the correlations it may have with other variables.

Causality usually cannot be directly observed, and so a long-standing goal of causality researchers has been causal discovery [12,13,14]: inferring the underlying causal model (or information about it) from observational data. Perhaps surprisingly, multiple causal discovery methods have been developed and successfully applied in different fields, such as biology, medicine, and economics, among others. Although we usually cannot directly infer causation from one observed correlation, causal discovery methods demonstrate that patterns of associations can be highly informative about causation.

This Special Issue presents recent advances in causal reasoning and causal discovery based on causal graphical models, including novel applications in different domains. It includes contributions from theory to applications in the following aspects:

**Theory:** A higher algebraic K-theory of causality is developed by studying the classifying spaces of observationally equivalent causal models; a *mutation sampler* (a rational process model of human causal reasoning that yields normatively correct inferences when sufficient cognitive resources are available but introduces systematic errors when they are not) is used to predict the existence of conditions in which Markov violations should disappear entirely.
**Causal reasoning:** Identification of causal outcomes is achieved when the available probabilities are more general, such as noisy observations or coarse-grained specification of the variables’ values; there are improvements in the identification of causal effects when some of the variables are functionally determined by their parents in the graphical model.
**Causal discovery:** An approach for dealing with insufficient data is developed that combines expert knowledge as a hard constraint and the structural prior gained via data learning as a soft constraint.
**Applications:** A novel technique based on causal models for business process management is devised; a framework that applies causal inference to canonical single-stage principal–agent problems in multi-agent contexts is developed; and a review of causal-based legal language processing is conducted.

Next we provide a brief summary of each article in this Special Issue.

## 2. Overview of Published Articles

Bejos et al. [15] apply causal graphical models to incentive design in a multi-agent context, which are central to economics and multi-agent systems. They introduce Causal Incentive Design (CID), a framework that applies causal inference to canonical single-stage principal–agent problems. Within CID, the operating rules of a principal–agent problem are formalized using an additive-noise causal graphical model. Incentives are modeled as interventions on a function space variable, which correspond to policy interventions in the principle–follower causal relation. This approach establishes a causal decision-making pipeline that enables commitment to a high-performing incentive in a single-shot game, supported by regret guarantees.

Friend et al. [16] investigate identification of causal quantities when the probabilities available for inference are those of the outcomes of more generic schemes than the usual joint probability distribution for the causal model’s variables, such as noisy observation procedures, or only coarse-grained specification of the variables’ values. Using process theories, they formulate graphical causal models as second-order processes that respond to such data collection instruments. Their main finding is that in the case of Markovian models, as long as the available instruments satisfy marginal informational completeness, the probabilities they produce suffice for identification of interventional quantities, just as those produced by perfect passive observations do.

The mutation sampler is a rational process model of human causal reasoning that yields normatively correct inferences when sufficient cognitive resources are available but introduces systematic errors when they are not. Rehder [17] uses the mutation sampler to predict the existence of conditions in which Markov violations should disappear entirely. Asking subjects to reason about a novel causal structure with nothing but generative causal relations (a cause makes its effect more likely) resulted in Markov violations in the usual positive direction. But simply describing one of four causal relations as inhibitory (the cause makes its effect less likely) resulted in the elimination of those violations. Theoretical model fitting confirmed how this novel result is predicted by the mutation sampler.

Causal discovery involves searching intractably large spaces; however, decomposing the search space into classes of observationally equivalent causal models can make it tractable. Mahadevan [18] studies the topological structure underlying causal equivalence to develop a categorical formulation of a transformational characterization of Bayesian networks. It characterizes Pearl’s structural causal models (SCMs) in terms of Cartesian cPROPs, where the morphisms that define the endogenous variables are purely deterministic. A higher algebraic K-theory of causality is developed by studying the classifying spaces of observationally equivalent causal cPROP models, constructing their simplicial realization through the nerve functor. A real-world domain modeling genetic mutations in cancer is used to illustrate the framework.

In the face of insufficient data, the accuracy and efficiency of causal discovery is greatly reduced, resulting in a false perception of causality. To solve this problem, Dang [19] et al. propose the use of expert knowledge as a hard constraint and the structural prior gained via data learning as a soft constraint. They develop a fitness-rate-rank-based multiarmed bandit (FRRMAB) hyper-heuristic that integrates soft and hard constraints into the learning process. For linear structural equation models, soft constraints are obtained via partial correlation analysis. The experimental results on different networks show that the proposed method has higher scalability and accuracy.

Chen and Darwiche [20] study the identification of causal effects, motivated by two improvements to identifiability that can be attained if one knows that some variables in a causal graph are functionally determined by their parents. First, an unidentifiable causal effect may become identifiable when certain variables are functional. Secondly, certain functional variables can be excluded from observation without affecting the identifiability of a causal effect, which may significantly reduce the number of needed variables in observational data. Their treatment of functional dependencies in this context mandates a formal, systematic, and general treatment of positivity assumptions, which are prevalent in the literature on causal effect identifiability and interact with functional dependencies, comprising another contribution of the presented work.

Conventional methods for process monitoring often fail to capture the causal relationships that drive outcomes, making it hard to distinguish causal anomalies from mere correlations in activity flows. Montoya [21] et al. introduce (CaProM), an innovative technique based on causality techniques, applied during the planning phase in business process environments. The technique combines two causal perspectives: anomaly attribution and distribution change attribution. It was validated with a industry dataset from the banking sector, comprising 562 activity flow plans. The study monitored causal structures during the planning and execution stages, allowing the identification of the main factor behind a major deviation from planned values.

Recent advances in legal language processing have highlighted limitations in correlation-based artificial intelligence approaches, prompting exploration of Causal Artificial Intelligence (AI) techniques for improved legal reasoning. Tritto [22] et al. examine the challenges, limitations, and potential impact of Causal AI in legal language processing compared to traditional correlation-based methods. Their findings reveal that while Causal AI frameworks demonstrate superior capability in capturing legal reasoning compared to correlation-based methods, significant challenges remain in handling legal uncertainty, computational scalability, and potential algorithmic bias. The scarcity of comprehensive real-world implementations and overemphasis on transformer architectures without causal reasoning capabilities represent critical gaps in current research. Future development requires balanced integration of AI innovation with law’s narrative functions, particularly focusing on scalable architectures for maintaining causal coherence while preserving interpretability in legal analysis.
